# Specifically formulated ketogenic, low carbohydrate, and carnivore diets can prevent migraine: a perspective

**DOI:** 10.3389/fnut.2024.1367570

**Published:** 2024-04-30

**Authors:** Angela A. Stanton

**Affiliations:** Stanton Migraine Protocol Inc., Anaheim, CA, United States

**Keywords:** migraine, hypersensory, ketogenic, LCHF, carbohydrate, salt, metabolic, carnivore

## Abstract

This article presents a hypothesis explaining the cause of migraines, suggesting that electrolyte imbalance, specifically a lack of sufficient sodium in the extracellular space of sensory neurons, leads to failed action potentials. The author argues that migraines are triggered when sodium channels fail to initiate action potentials, preventing communication between neurons. The article discusses the evolutionary perspective of the migraine brain, stating that migraineurs have a hypersensitive brain with more sensory neuronal connections, making them more reactive to environmental stimuli and in need of more minerals for the increased sensory neuronal communication. Since glucose is often used to reduce serum hypernatremia, it follows that a high carbohydrate diet reduces sodium availability for use in the brain, causing an electrolyte imbalance. Low carbohydrate diets, such as ketogenic, low carb-high fat (LCHF), and carnivore (all animal products), can be beneficial for migraineurs by reducing/eliminating carbohydrate intake, thereby increasing sodium availability. In support, many research papers and some anecdotal evidences are referred to. The article concludes by proposing lifestyle modifications, such as dietary changes and sodium intake management. These will provide migraineurs with a long-term healthy metabolic foundation helping them to maintain strong nutritional adherence and with that aiding continued proper neuronal functioning and migraine free life.

## 1 Introduction

Migraine is one of the top 10 most disabling conditions, leading to much suffering and negative lifestyle consequences, including loss of work (1). Current treatments available for migraine headaches are not effective; most of them do not work, or if they do, they have very serious side effects. Migraine medications aim to reduce or prevent headache symptoms but do not stop or prevent the migraine itself. Headache is just one very common but nonessential symptom of migraines. There is no scientific consensus on the cause of migraine. Disparate research areas are heading in different directions, suggesting various mechanisms. Not only is migraine not understood, but some of the medications prescribed for it have unknown mechanisms of actions, like Flunarizine (Flumig or Sibelium) (2), Amitriptyline (Elavil) (3), Levetiracetam (Keppra) (4) and many others. Migraine is a “black box” condition. Yet interestingly these and most other drugs for migraine do tap into the same areas this paper covers, only, as the reader will see, they hinder rather than support healthy brain activity.

That migraine is little understood can be seen by the variety of studies that try to define what it is and what it is caused by. Researchers usually look to food triggers (5), environmental variables like the weather (6), vascular conditions, or hormonal changes (7), and even suggest that migraine is predominantly a “woman’s disease” (8) and therefore female hormones are often blamed. While the majority of migraineurs are female, a large percent of them is menopausal or post-menopausal and, of course, how do we account for the small but still present male and child populations with migraines? And how do we account for those females who do not get migraines from their hormonal variations? In fact, in the years prior to puberty, migraine is more common among boys than girls (9), and colic is suspected to be an infant migraine presentation (10). Clearly, while hormones may make things worse in migraine, they cannot possibly be the cause of it.

While other suggestions are more general in nature, many of them can be eliminated from further consideration by the available evidence. Migraines have also been associated with anxiety and other psychiatric disorders (11), vascular disease (disease of the blood vessels), cardiovascular disease (disease involving the heart) (12, 13), MTHFR gene polymorphism (14), contraceptives (15), stroke (16), increased matrix metalloproteinase activity (17), oxidative stress (18, 19), neuropathy (20), blood-sugar level variations (21), maladaptive stress response (22), and metabolic disease (17, 23, 24). And with each field so narrowly defined, it is hard, if not impossible, for specialists within any one of these fields to look outside and find patterns of similarities and differences, so that many areas of research could be combined, and conclusions drawn.

Some researchers suggest that migraine is not inside the brain but is extracranial, and is associated with arterial dilatation (25). While extracranial dilation may or may not be associated with migraine, it most certainly is not the cause of it. Imagining studies show changes inside the brain right before or during a migraine. Examples are: cortical hypoactivity that is characterized by a decreased level of neuronal pre-activation excitability (26) or neuronal hyperexcitability (27, 28), structural abnormalities of the brain (29), brainstem dysfunction (30), white matter abnormalities and/or infarct-like lesions and/or volumetric changes in gray and white matter regions (29), neurogenic inflammatory responses from CGRP releasing trigeminovascular network of neurons (31), abnormal function of receptor channels of sensory neurons in some cortical areas that stimulate perivascular intracranial nerve fibers (32), structural and functional brain alterations (33), occipital cortex hyperexcitability (34), neurotransmitter and neuromodulator metabolic abnormalities (35), a spreading depression-like neuroelectric event during migraine aura (36) and in migraine without aura (37), hsCRP-measured cerebral white matter hyperintensities (13), and many more.

A number of research papers on migraine discuss the differences between neuronal plasticity and variations in excitatory vs. inhibitory behavior of the neurons (38, 39), as well as differences in functional connectivity between the brain of migraine sufferers and non-sufferers (40), as well as yet unexplained white matter differences (41). Migraine and seizure share many of their features. The Epilepsy Foundation suggests that those having an epileptic seizure disorder are twice as likely to also have migraines. Misdiagnoses are frequent because the symptoms are so similar (42, 43). In the case of epileptic seizures, the “seizures are generated by hyperexcitable and hypersynchronous neuronal firing that leads to the rhythmic recruitment of large populations of neurons. A seizure is triggered when a sufficient number of neurons synchronously depolarize and generate action potentials” (43), and this is quite similar to what happens in migraine, only the neurons do not depolarize synchronously but in a wave, which is referred to as Cortical Spreading Depression (CSD). Although it is still debated whether CSD even exists, and if it does, what its role may be (34), here we will emphasize the role of CSD, which we believe to be a crucial and characteristic phase of migraine. CSD is not exclusive to migraine, it is also part of seizure, brain injury, and other conditions (44).

Looking at any of the research areas, none provides a comprehensive explanation of how and why what they found occurs, and how it generates migraine with or without headache. A comprehensive theory must explain the major manifestations of the condition, must generalize the phenomena to similar or related entities, and must include hypotheses that can be supported, refuted, or improved on (45), to explain how and why a migraine starts and why it often leads to pain. It is clear that not only is the cause of migraine elusive, but also the manifestation of migraine is not understood. In addition, although migraine need not have pain accompanying it (46), it appears that almost all research is following the headache aspect of migraine, which suggests that whatever they find could not explain what “migraine” is; they only aim at preventing migraine headaches.

With little understanding of what migraine actually is, no wonder that good and reliable prevention measures or treatments are not available. I found that even the diagnostic practices are questionable. For example, migraines always come with prodromes, yet prodromes are hard to define or recognize by the migraineurs themselves. An experiment, using electroencephalogram, detected prodrome as a higher complexity of brain activity in patients who were in the preictal phase (prodrome phase) than in patients during the interictal phase (the actual migraine) (47). Understanding prodromes and helping migraineurs discover when they are in a prodrome phase can help them avoid a migraine. Doctors rarely if ever ask if the patient experiences prodromes. Ignoring this means that a doctor may diagnose some other form of headache, such as cluster, sinus, cervicogenic, stress, occipital neuralgia, optic neuritis, or idiopathic intracranial hypertension as migraine.

Existing guidelines are not clear and are also ignored or overruled by physicians. The International Headache Society is the main authority on headache types, and migraine headache is defined by it as a unilateral pain (48). Yet in a large percent of the literature and online guides—such as Medscape (49) or Merck Manual (50) and other scientific literature (51) this definition is not followed and even stated that migraine can also be bilateral. There are many other areas where confusion exists in the definition of what a migraine headache really is. Per the International Headache Society migraine is a primary headache, meaning it is not caused by any preexisting condition. Nevertheless, much literature informs us (and also reported to me by a large number of people) that doctors often diagnose any “big headache” as migraine headache, even when it is caused directly by a traumatic brain injury or some other health condition (52). There is a misdiagnosis crisis of migraines.

Many studies discuss how well epileptic seizures can be lessened and or prevented by lifestyle modifications, specifically by the ketogenic diet (53–56). Given the similar pathophysiological nature of seizure and migraine, the benefits of the ketogenic diet need to be examined, not just to see if it works to lessen migraine headache frequency or eliminate migraines completely, but to also give us clues as to the cause of migraine. It is likely that if we can reduce the incidence of a condition by a particular treatment, the treatment itself can be reverse engineered to shed light on the cause.

As we have seen, there is no widely accepted, comprehensive definition of migraine. One of the confounding factors is that most migraineurs have multiple “types” of migraine symptoms. Literature separates migraines into “migraine types” based on symptoms and assumes that once a person is, for example, a hemiplegic migraineur, that person will have all her migraine hemiplegic. Based on the numerous cases that I have come across—including my own migraines that have contained a number of different migraine types from classic aura to scintillating scotoma, to complex migraine (without aura), to light hemiplegic (arm tingling and loss of strength, droopy eye)—I can firmly state that migraine type based on symptoms is not a stable, life-long determinant.

While this paper will cover all migraine types, after identifying the root cause, it will become clear that all migraine types are just one thing: migraine. What makes them appear different is the area of the brain being affected. The cause is the same in all migraines, no matter what their symptoms and where in the brain they start. Based on the brain differences between a migraineur and a non-migraineur, if we place them into the same room with a specific light, odor, and sound setting, they will sense a completely different environment around them. The migraineur will find the light brighter, the sound louder, and the odor stronger. What this suggests is that migraineurs are not sensitive to bright light, but they see regular light brighter, they hear regular sounds louder, and smell regular odors stronger. Their brain is more “environment adapted.” As was introduced earlier, this was an evolutionary advantage in human ancestral life, but it has become a burden in our modern bright, loud, odorous lives full of excitatory stimulants that play havoc with a brain brimming with sensory neurons that overreact to them.

## 2 The cause of migraine

I first define the cause based on my hypothesis, and then proceed to show why and how it is correct. At the same time, I aim to bring up as many opposing arguments to it as I can think of.

### 2.1 The hypothesis

The cause of migraine is an electrolyte imbalance, specifically, not enough sodium in the extracellular space of the sensory neuron (s) to initiate action potential. Action potentials spread information in the nervous system to connected neurons and propagate commands to the periphery. If the action potential fails at any Node of Ranvier, the neuron’s communication is stopped, and instructions never reach their intended target. The neuron moves back into resting state with voltage-gated sodium channels closed. The symptoms resulting from blocked or malfunctioning sodium channels or insufficient sodium at the channels are: seizures, altered mental status, hypotension, prolonged QRS (ventricular depolarization in the heart), a terminal R-wave in lead to aVR (57), edema, swelling of the brain, coma, death, and I argue that this list should also include migraine.

We should ask why the brain would ever be short of sodium to the extent that the neurons cannot function as a result of failed action potentials. Common causes of hyponatremia are dehydration, vomiting, diarrhea, too much fluid consumption (water toxicity), type 2 diabetes, hyperglycemia, hyperkalemia, malabsorption disorders, kidney failure, heart failure, cirrhosis, diuretics, cerebral hemorrhage, subarachnoid hemorrhage, Guillain-Barré syndrome, head injury, brain tumor, meningitis, certain medications, hypomagnesemia, hypocalcemia, vitamin D deficiency, and there are many more (58).

To ensure properly functioning osmolality in the brain the right amount of sodium, chloride, and potassium must be present. At equilibrium, extracellular osmolality equals intracellular osmolality, and the net movement of water across the cell membrane is zero. When the extracellular sodium concentration is reduced, hypo-osmolality and hypotonicity will ensue as the water flows from the extracellular space into the intracellular area. The water movement into the neuron causes its swelling. In the brain, even minimal changes in the intraneuronal volume (specifically swelling) leads to dramatic symptoms due to the lack of space (59). Neuronal adaptation to hyponatremia involves movement of electrolytes from inside the cell to the extracellular area. Within the first hours of hyponatremia, there is a significant decrease in the intracellular content of sodium, chloride, and potassium (60, 61). The kinetics of brain electrolytes depletion during acute hyponatremia have been studied and described. After 3 h of hyponatremia, brain depletion in electrolytes reaches a plateau, and the depletion of sodium is believed to be primarily from the cerebrospinal fluid, which occurs together with intracellular depletion of chloride faster than the intracellular depletion of potassium (62). In total, the brain can lose no more than 18% of its ion content (59). It is expected that by the time the mechanisms behind electrolyte loss are exhausted, severe continued hyponatremia will inevitably cause significant brain edema. Hyponatremic encephalopathy has many very similar symptoms to migraine: headache, nausea and vomiting, fatigue, confusion, and loss of balance, pointing to similar pathophysiology of the two conditions.

Furthermore, renal sodium wasting is a common feature of migraineurs. An early study showed that migraineurs excrete 50% more sodium in their urine than non-migraineurs (63). And hyponatremia is the most common cause for electrolyte imbalance in the brain (64). It is important to elaborate that the brain may suffer a hyponatremic event for reasons other than dehydration. Hyponatremia may be caused by the foods we eat as well. A study showed that for every 100 mg/dL increase in serum glucose concentration, the average decrease is serum sodium is 2.4 mEq/L (65), but this reduction in serum sodium is not linear. With the ranges of normal sodium levels of 135–145 mEq/L, a 2.4 mEq/L drop can easily tilt the person toward serious hyponatremia by simply eating lots of carbohydrates (66). In fact, mortality rate increases when serum sodium levels drop from 139 mEq/L to 132 mEq/L (67). Since hyperglycemia is a causal factor in hyponatremia, the reduction of hyperglycemia will prevent a dangerous drop in serum sodium levels.

Hypernatremia is less likely to occur, and if it does, it is less likely to cause any trouble in the brain, because it induces the movement of water across cell membranes in the opposite direction from hyponatremia (68). Hypernatremia induces hypertonicity and causes transient cellular dehydration (69, 70). Sustained hypertonicity promotes the accumulation of organic osmolytes (e.g., glutamate, taurine, and myo-inositol) and these adaptive changes thereby pull water into the cells and restore the cell volume (71, 72). Therefore, chronic hypernatremia is much less likely to provoke neurologic symptoms.

At this point it is important to introduce the actual anatomical and physiological differences that distinguish the migraine brain from the brain of a person without migraines.

### 2.2 The evolution of the migraine brain

The sensory neurons in the brain of a migraineur have more connections (73) than in the brain of those without migraine and these connections themselves also differ from the norm (74). Migraine brain seems to always be “on,” as migraineurs have only nominal changes in voltage between states of action potential and resting potential (75–77). Clearly, the brain of a migraineur is anatomically different from the brain of a non-migraineur, and as a result, it has been called the hypersensitive brain (78). The sensory neurons in mammals evolved during periods of high vulnerability levels, when vigilance was a major component of survivability, and the heightened sensory sensitivity presented a survival advantage.

Limited studies exist on the evolution of specific sensory networks in the brains of mammals (79), and these studies do not compare modern humans to other mammals in their native wilderness, where predation presents a risk to life, and where heightened sensory organs provide fitness for survival. Some studies that compare some of the human sensory organs to those of other primates conclude that humans have lost some of their ability to smell relative to other primates (80). The hypersensitive brain had to be the original standard but by now the majority of humans have adapted to a lack of danger from predatory animals and to a measure of predictability and safety of their environment. A great number of the human population have also adapted to city-dwelling and the associated noises, odors, and lights with excitation inhibition of their sensory neurons.

Why females and children are more vulnerable can also be explained by the hypothesis. Generally men were the hunters, carrying weapons, and the success rate of a predator killing a hunter was likely quite low. By contrast, women and children were left behind to gather—squatting or bending down without weapons, they are the ideal pray for a predator. As a consequence, this group developed and retained stronger sensory organ systems, especially for the time of their heightened vulnerability. Before puberty, more boys have migraines than girls (81). Boys became hunters at or shortly after puberty and correspondingly we see most boys losing their migraines after puberty (81). In our modern Western civilized world humans have no need for keen sensory organs against predators, and so it makes sense that such an energy sink would have devolved by adapting to a less sensitized human lifestyle with reduced ability to sense.

However, a group of people, the migraine sufferers, appear to lack this adaptation. Comparison studies of the sensory neurons of the sensory organs are lacking, but what we do know is that migraine is initiated by odor, light, sound, taste, and touch, suggesting that migraineurs form a subpopulation of humans who are not properly adapted to modern life full of odors, noise, light, and other potentially overstimulating factors. The explanation for this is the lack of proper neuronal inhibition that some studies do show (82–84).

It is well understood that the migraine brain responds to stimulation differently from the norm; it is easily overstimulated and has difficulty dealing with a hyperexcited state (78). Excitation in the brain is defined as communication between neurons via neurotransmitters, which move through each neuron by electricity created in the axon at each Node of Ranvier and rushes through the spike train to the axon terminals (85). An average neuron has a Node of Ranvier at about every 350 μM (86), and at each one there are approximately 700,000 voltage gated sodium channels (87). These sodium channels are responsible for the initiation of the electricity by generating action potentials, which then can move to the next Node of Ranvier by saltatory conduction (88) and all through the axon of the neuron to the axon terminals.

Neuronal excitability depends on membrane potential that can be altered by neurotransmitters released at synapses. Membrane potential is created by selectively permeable ion channels in the membrane. Altering the membrane potential creates a current across the membrane. However, every action potential is either excitatory or inhibitory. Excitatory currents are those that prompt one neuron to share information with the next neuron through an action potential that leads to the release of excitatory neurotransmitters, while inhibitory currents are action potentials that send inhibitory neurotransmitters into the synapse. In addition, the action potential itself also differs between excitatory and inhibitory release. Disruption of the balance between excitatory and inhibitory inputs is one likely cause of diseases marked by bouts of abnormal neural activity (83).

In the case of inhibition, it is an inhibiting neuron that is used for activation in order to release GABA, an inhibitory neurotransmitter. Its activity reduces the amount of neurotransmitter released into the presynaptic area, and with fewer Ca^2+^ rushing into the neuron, less neurotransmitter is released into the target synapse. There is also postsynaptic inhibition, where the dendrites of the postsynaptic neuron cancel some of the incoming signals, receiving fewer average stimulus, too small to start an excitatory state.

Most of the sodium channels in the Nodes of Ranvier are Nav1.6 sodium channels. These channels are responsible for the generation of the action potentials that initiate the electricity that must move through the axon to either excite or inhibit. There is a threshold number of these sodium channels that must be met if the action potential is to be successfully generated. Presynaptic action potential failure prevents transmission to postsynaptic neuron, stopping the spike train (89, 90). Since the brain of the migraineur has altered sensory neuronal connections and more of them (33, 91) firing in what is referred to as hypersynchrony (92) or more commonly in migraine as cortical spreading depression (CSD) (93), a lot more sodium and ATP is used by the brain of a migraineur than that of a brain of an individual without migraines.

Myelin is a cholesterol and fatty acid rich substance that serves as a specialized insulation sheath around the axons in the nervous system, facilitating axon signal conduction by enabling saltatory conduction. Damage to the myelin sheath can inhibit saltatory conduction and prevent neuronal transmission. Myelin sheath damage is commonly seen on brain MRIs of migraineurs (94, 95). “Myelin can decrease the capacitance by a factor of up to 1,000” (39) suggesting that damage to the myelin increases the need for charge, necessitating a larger number of sodium ions entering through more sodium channels.

Several academic articles refer to migraine as channelopathy (96–99). Channelopathies are diseases associated with defects in ion channels, either genetic or acquired (100). A channelopathy may cause an abnormal “gain of function” (such as myokymia (101) and ptosis (102), that are commonly missed by practitioners, even though they are often associated with migraine), or an abnormal loss of function (such as weakness or numbness) depending on whether loss of channel function leads to excessive excitability or to lack of excitability (103). In the case of migraine, several ionic channels are genetic variants, including the sodium-potassium ATPase (Na^+^/K^+^/ATPase) channel, which is responsible for resetting the membrane potential to resting state (104) to rebuild the saltatory conduction. In addition, visiting the human genome database (105) and looking at the currently discovered genetic variants associated with migraine, in order of relevance of the variant to migraine, the first three most relevant variants out of 3,866 to date are the ATP1A2 (Na^+^/K^+^/ATPase Subunit Alpha 2), CACNA1A (Calcium Voltage-Gated Channel Subunit Alpha A), and SCN1A (Sodium Voltage-Gated Channel Alpha Subunit 1). These are the three main voltage-gated ionic channels whose proper functions are most critical to neuronal communication, and which are variant.

As noted earlier, an important aspect of migraine is CSD. During CSD, a large ionic shift with the redistribution of ions between intracellular and extracellular compartments takes place in the brain (106) intermixed with a pH shift (107, 108). The ionic shift appears to increase the sodium availability, and with the changes in cerebral pH a large increase in lactate is seen in the brain (107). The neurons in the brain are able to use lactate fed to them by the astrocytes instead of glucose. The reason for the increased lactate is possibly associated with a sugar crash (reactive hypoglycemia), which may follow a carbohydrate meal. Studies show that dietary carbohydrates cause fatigue and brain fog as part of reactive hypoglycemia (109). These events are described in great detail elsewhere (106), but in brief: As cells lose energy resulting from a sugar crash and/or hypoxia, voltage-gated pumps that normally move ions into and out of the cell fail or operate in reverse. These induce a rapid efflux of K^+^ from intracellular space, causing an increase in extracellular K^+^. The rapid rise in extracellular K^+^ elicits neuronal excitation, followed by excessive depolarization and a period of electrical silence during which the potential at the brain surface becomes negative. Ca^2+^ ions flow in as the depolarization opens voltage gated Ca^2+^ channels and extracellular Ca^2+^ falls to abnormally low levels. Na^+^ and Cl^−^ enter neurons. Water follows passively, driven by the influx of Na^+^ and Cl^−^, which greatly exceeds the efflux of K^+^. The extracellular space is reduced, and local intracellular edema ensues.

In case of migraine brains, because of the hyperexcited state of the sensory neurons, more fuel is needed. The amygdala initiates a fight-or-flight response, sending a message to the adrenal glands to release epinephrine (adrenaline), which in turn triggers many functions. One of them is the release of glycogen for more energy in order to facilitate the extra work and the need for recovery of the brain. The glial cells, primarily the astrocytes, store some small amounts of glycogen to ensure constant glycogen supply in the brain in order to prevent a critical sugar crash for short amounts of time. However, as glucose enters a neuron large amount of sodium leaves (110). This causes localized extracellular edema and reduction in the intracellular Na^+^ levels, increase in the intracellular K^+^ levels relative to Na^+^, closure of the voltage-gated sodium channels, and ultimately failed action potential.

**Figure 1:**
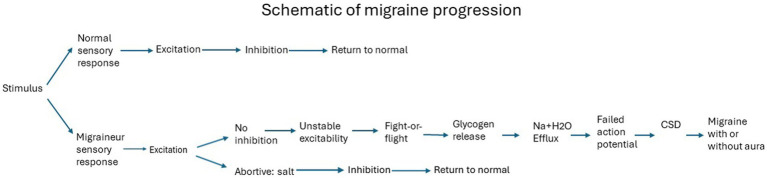
Migraine Progression.

And this is where we reach the state when migraine starts. To visualize the differences in response to the same stimulus by a migraineur vs. a non-migraineur, the following schematic demonstrates the summary of events. The specific stimulus elicits the conditions for a migraine (111), generating unstable excitability (112), leading to migraine following the order of events as described above (Figure 1).

Furthermore, while all people will experience similar efflux of sodium and water from neurons in response to glucose, a sodium imbalance will not trigger other conditions with similar frequency or with the same symptoms. For example, epileptic seizures may also be caused by electrolyte imbalance and reduced sodium availability (113), but the symptoms of seizures are very distinct and easy to tell from migraine. Other clinical conditions, such as dehydration, renal failure, diabetes insipidus, or sodium wasting (63), all can cause serious modifications of plasma osmolality and electrolyte imbalance, causing alterations in brain metabolism and function (114). The driving force behind the extreme sensitivity to these osmolality changes in electrolytes in migraines is associated with the anatomic differences of the sensory neurons in the brain of migraineurs. Migraineurs have excess sensory neuronal connections (73) and that means more action potential is needed to work with the incoming sensory stimulus.

One may ask about the importance of other electrolytes, such as potassium, calcium, or magnesium. How might they connect to migraines? Most of the potassium is inside the cells whereas most of the sodium is outside of the cells and potassium is not affected by glucose entering the cells because potassium is not a glucose cotransporter. Potassium levels are modulated by many conditions, such as kidney failure, diabetes mellitus, adrenal disease, angiotensin-converting enzyme inhibitors, angiotensin receptor blockers, and potassium-sparing diuretics (115), but these conditions are not associated with migraines and so are not discussed in this paper. Magnesium does not leave because of glucose entering the cells and so does not affect electrolyte osmolality at all. And lastly, while more calcium is needed as a result of more neurotransmitter delivery into the synapse, calcium is not affected by glucose entering neurons either. The only mineral that is actively affected is sodium. Sodium is an active glucose cotransporter in the body as well as in the brain, where SGLT1 and SGLT3 cotransporters are used.

### 2.3 The nutrition connection

The availability and variety of nutrition for humans can be followed by moving back in the evolutionary timeline. For most of human evolution humans mostly consumed animal products if they could, with some carbohydrates mixed in as necessity or seasonally desirable. For 99% of human history, humans had a hunter-gatherer lifestyle (116). It wasn’t until about 12,000–15,000 years ago that the diet of some human groups started to include larger amounts of plants filled with carbohydrates. In addition, the ancestral human lived without loud smelly cities with cars, and flashing lights, and so they did not live in a constant hyperexcited sensory state, where electrolyte imbalance is highly likely. Consequently, a diet that reduces electrolyte imbalance should be beneficial for migraine sufferers.

The question then becomes, what may a migraine sufferer eat that would affect her hypersensitive sensory neuronal brain in a way that prevents the development of a migraine? Can a change in lifestyle drive a change in migraines? And if so, how does a new way of eating prevent migraine and migraine headaches?

### 2.4 Carbohydrate and migraine

As we have just concluded, the hypersensitive sensory neuronal migraine brain possesses a high number of connections, that transmit more signals because of the higher number of registered environmental excitatory inputs. As a result, the brain of a migraineur has not only increased energy requirements in terms of fuel (ATP) but also in sodium to operate the increased electrical activity of this hypersensitive brain. The brain uses glucose, lactate, and glycogen as its primary fuel for about 25% of its energy need, as these are the exclusive fuels for astrocytes. While some neurotransmitter generation do require glucose, most may be generated by the use of ketones (117).

An interesting phenomenon occurs because of increased glucose use by the mitochondria, due specifically to the pyruvate conversion process in the form of excess reactive oxygen species (ROS). A study showed a metabolic collapse in the hippocampus—the area of the increased ROS—resulting in ionic channel malfunction and CSD-like depolarization (118), which is precisely what precedes a migraine. CSD is not the exclusive property of migraine; many other neurological conditions also exhibit it, including mechanical brain damage, electrical stimulation, hypo-osmolarity, hyperthermia, chemical agents such as potassium, the neurotransmitters glutamate and acetylcholine, acute hyperexcitability, sodium pump inhibitors, hypoglycemia, hypoxia, ischemia, and it can also be induced by noxious odor challenges (119–122). CSD is a common occurrence and seeing it preceding a migraine is not surprising. One may envision CSD as a brain-driven self-rescue system to reallocate available resources to neurons across the brain. In fact, a study on rats concluded that CSD can also be induced by exercise and the CSD leads to beneficial effects in cerebrovascular system functions and increased cerebrovascular stability (123).

When the brain does not get enough glucose, the neurons will switch to ketone use. However, it is seldom discussed if a brain, given plentiful glucose and ketones at the same time, would prefer to use glucose or ketones? Since glucose is considered to be the prime fuel for the brain by most academic literature, the assumption is that as long as glucose is provided for the brain, it will use glucose. But this is incorrect. A study showed that the brain will preferentially use ketones over glucose even when both are present (124), where exogenous ketones were supplied to otherwise glucose-rich brains that had degenerative diseases caused by glucose metabolism difficulties in the brain. This paper suggests that a migraine brain seems to have glucose metabolism difficulties. This leads to the important point that it is not whether glucose is available or not, but rather can the brain metabolize glucose or not? Experiments on rats show that ATP is increased when ketones are used by the brain (125). This study further suggests that with ketone use by the brain, neuronal stability is followed.

Studies on human fetuses while in the mothers’ womb show that the fetus’ brain selectively uses ketones, yet clearly the fetus’ brain is well endowed with the opportunity to use glucose if it wishes (126, 127). And babies retain metabolic flexibility for a number of years as they come in and out of ketosis based on their feeding schedule (128). Clearly, such stability is what the brain could benefit from in the case of migraine (and other brain-diseases) as well. And as mentioned repeatedly earlier, glucose entering neurons causes an efflux of sodium, contrary to the increased requirements, creating instability. The logical conclusion is that a reduction of carbohydrate consumption and an increase of ketones and sodium in the brain are likely beneficial for migraine prevention.

The cascade of events leading to the inability of firing action potentials can be prevented by increased sodium (129–131) and reduced carbohydrate consumption. The consumption of carbohydrates is not essential because gluconeogenesis by the liver provides the necessary glucose to all organs as needed. The primary fuel for the body is fat, not glucose (132–135). The primary preferred fuel for the brain is ketones, as demonstrated earlier. Diets low in carbohydrate, such as the ketogenic, low carb-high fat (LCHF), and carnivore diets (136, 137), as well as consumption of appropriately higher levels of sodium (130, 138) are beneficial. Studies show that fatty acid utilization for fuel in the brain is key for repairing neurodegenerative disorders (139). Ketone bodies are an efficient fatty acid fuel that can compensate for the deficient glycolytic metabolism of the migraine brain (140).

The ketogenic diet has been a much-researched approach in many neurological conditions (141, 142) because the brain is very specifically adapted to use ketones as fuel instead of glucose (143). Many studies have focused on the ketogenic diet especially for epilepsy (144) but some studies show its benefits in migraine as well (145). However, numerous concerns have been voiced because of the temporary side effects of converting from a high carbohydrate diet, where the brain and much of the body use glucose as its primary fuel, to the ketogenic diet, where the brain’s and some organs’ primary fuel changes to ketones. While the brain appears to make the change from glucose to ketones quite easily (146), it is not so easy for the body for some people. There are two primary issues: the ketogenic diet is restrictive, and some people find it difficult to adhere to and there is an adaptation period that can cause discomfort for some.

While the ketogenic diet is restrictive, the extreme carbohydrate sensitivity of migraineurs gives near immediate feedback about the benefits or harms of dietary choices. If the migraineur gets a migraine each time a high carbohydrate meal is consumed and remains migraine free after a ketogenic, LCHF, or carnivore meal, the migraineur will happily accept the new regimen.

Some of the known physiological difficulties are grouped under the term “keto flu,” represented by fatigue, headache, and gastrointestinal difficulties associated with the body converting to ketone use. A similar condition is referred to as “fat adaptation” in athletes who restrict carbohydrates in order to train for a competitive event a couple of weeks later, because the body using fat for fuel can become stronger and important glycogen stores are spared (147). The reason for keto flu is debated (148), but most agree that it affects a large part of the general population when starting the ketogenic diet (149). It is likely associated with the loss of fluids and electrolytes, specifically sodium, as insulin drops to lower levels in the body quite drastically after the reduction of carbohydrates. With high insulin, the kidneys recycle sodium and retain excess water correspondingly (150). It is also important to replenish lost electrolytes, because the ketogenic diet is a fasting mimicking diet (151), and the increased use of glucagon in response to fasting reduces both sodium and water (152).

Additionally, keto adaptation is not the same for each person. Some people enter the state of ketosis very soon after starting the ketogenic diet and without keto flu. Since comprehensive research is lacking in this area, we cannot forecast how long such adaptation will take and when any benefits of the ketogenic diet can be realized for a given candidate. In my limited experience I found that the benefits accrue sometimes from as little as a couple of weeks to as much as many months, depending on how long the person had migraines, what kind of medications were taken, how damaged the metabolic health of the individual is, and also the age of the individual.

A critical point is the potential interactions between certain medications and the ketogenic diet. For example, Topiramate (Topamax) label lists serious interactions with the ketogenic diet as it may lead to kidney stones. Although other drugs have not been labeled with warnings, caution must be taken, and frequent de-prescribing may be necessary. Patients on the ketogenic diet find their insulin and weight dropping very fast and medication doses correspondingly need to be reduced. The ketogenic diet has shown blood pressure reduction (153), the reversal of kidney disease (154), reduction of HbA1c and increase of HDL (155, 156) and even the reversal of type 2 diabetes (157). Many thousands of anecdotal reports point to great success with the ketogenic diet as well as with the lesser studied carnivore and LCHF diets, providing an incentive for others to try them.

Our next questions are: Can we remove carbohydrates from the diet of migraineurs safely? Can we increase the availability of sodium to the brain of migraineurs safely and what is the best way? Will these steps help reduce or prevent migraines?

### 2.5 The safety of salt

There are many research papers showing results in applying ketogenic or other low carbohydrate diets to migraine, but studies do not exist about adding extra salt. In fact, there is quite a bit of concern surrounding the increase of dietary sodium. Here I address some of the issues.

Strong anecdotal evidence suggests that increasing salt, specifically when taken with water (as opposed to eating more salt with food) in order to increase sodium availability in the blood for the use of the brain, is beneficial. Electrolyte drinks are sold in stores and research shows that they have beneficial effects in hydration over water (158). Studies show that migraineurs who consumed more salt reported fewer headaches (129). Hypertension and hypotension both are considered to be comorbid with migraine and therefore it is suggested that the cardiovascular risk profile is higher in migraineurs (159–162). However, I previously questioned the perceived cardiovascular risk aspect of migraine (163) and it is very easy to overlook that the medications migraineurs are placed on often cause cardiovascular diseases on their own. Might it be that migraineurs end up with hypertension and increased cardiovascular disease as a result of the medications they are taking for migraine? For example, NSAIDs are the choice for over-the-counter use, and they are well-known to cause cardiovascular problems (164). Propranolol (propranolol hydrochloride) is a frequently prescribed medication for migraine, although it is actually a strong heart medication with significant cardiovascular health concerns (165). Triptans are the most often prescribed medications for migraine and there are very serious concerns with respect to the cardiovascular and heart damage they cause (166). Various SSRIs and TCAs are often prescribed for migraine as well. While SSRIs are deemed safer than TCAs in terms of cardiovascular profile, they are not completely safe (167). CGRP inhibitors are the latest class of medications recommended for migraine and because they are so new, less information is available in published literature. But some academic articles point out that the cardiovascular system has CGRP receptors in order to initiate vasodilation. When the CGRP receptors are inhibited, vascular damage and associated dangers arise (168, 169). Given the many medications prescribed to migraineurs and that the evaluation of cardiovascular disease associated with migraine often does not include a questionnaire for what medications migraineurs take, I believe that it is irresponsible to suggest that migraineurs are generally more susceptible to end up with hypertension and cardiovascular disease, absent any medications.

While there is much general concern about increased dietary sodium and its association with hypertension, the physiology of sodium use by the body and the method of elimination of the excess sodium does not give rise to hypertension concern in the metabolically and thereby cardiovascular-healthy, individuals, and, in fact, the opposite is true: a reduced sodium diet leads to hypertension (170, 171). In addition, reduced sodium diets are now understood to cause insulin resistance and cardiovascular disease (172, 173). And a study showed that even in the case of subjects with salt sensitive and salt resistant hypertension, while their systolic pressure dropped minimally on a reduced sodium diet, their insulin resistance markedly increased (174). It is also interesting to note that while most people in pain tend to have an increase in blood pressure, a study showed that migraineurs suffer from hypotension before, during, and shortly after a migraine attack (175). This is further validation that migraine is associated with hypovolemia due to electrolyte imbalance and loss of sodium. And hypovolemia is a consequence of inappropriate hydration and high carbohydrate consumption. And lastly, a study injected saline directly into the arterial vein of the brain and found that all subjects experienced significant relief, with a large percent having complete pain relief (176).

Therefore, even if the consumption of salt may increase blood pressure in some people, given the hypotension migraineurs exhibit before, during, and after a migraine episode, an increase in blood pressure would clearly be welcome. Additionally, one may ask: if salt increases blood pressure, why is intravenous saline the first line of treatment (for migraine as well as for many other conditions) in most emergency departments (177, 178)? And would not an increase in salt (an essential mineral) be safer than the taking of unsafe medications with lots of side effects?

Salt in water is an electrolyte and it heads straight to the blood since it is not “food” *per se*. And since most of the sodium in our body is in our blood and outside of the cells, drinking salt with water is the fastest and safest way to regulate the sodium amount in the blood. There are no human experiments on salt in food vs. water, but a mouse study shows that the pathways of sodium absorption are different in food from water (179). We excrete salt both via the kidneys and also via feces, although much of what is excreted in the feces is reabsorbed by the colon (180).

### 2.6 The safety of low carb diets

The low carbohydrate diets have initiated quite a controversy over the past few years. However, by now there are dozens of clinical trials associated with research on low carbohydrate diets. Not only are they safe, but these trials show them effective in helping, and in some cases reversing various illnesses, or at least putting a particular condition into remission. Specifically weight loss, cardiovascular health, type 2 diabetes, and many neurological conditions, such as epilepsy, Alzheimer’s disease, Multiple Sclerosis, Parkinson’s Disease, Schizophrenia, and many more conditions have been shown to strongly benefit from a low carbohydrate diet (157, 181–187).

Overwhelming anecdotal evidence suggests that the application of any of the low carbohydrate diet forms: LCHF, ketogenic, and carnivore, provided they are well-formulated for health with sufficient protein and fats, should be helpful in preventing migraine, but the reason why it is so, is often misunderstood. For example, a small trial concluded that the ketogenic diet is likely beneficial because it helps people lose weight and migraine sufferers are overweight (188). Interestingly there are many studies pointing to migraineurs being overweight (189, 190), yet my experience in working with thousands of migraine sufferers from around the world is that they are not overweight—in fact many are underweight as a result of being unable to eat while they are so often in pain. Regardless, weight loss on its own is not likely to lead to the reduction of migraine given that it is a genetic condition of ionic channel variants and the brain’s glucose intolerance (191). Rather, with the help of the hypothesis laid out in this paper we can understand that it is the reduced carbohydrates in the ketogenic and other low carbohydrate diets, especially the carnivore diet, and the increased salt that provide relief for migraine sufferers.

Let me bring a couple of specific anecdotal evidences, where the benefit of the diet change and especially the use of salt in water was very specifically the cause of the migraine free life.

In one example, a marathon runner approached me for help. She was not overweight and appeared metabolically healthy but she would run 10 miles daily for practice and always end up with a migraine. She was also suffering from monthly hormonal migraines with her cycles. Knowing that estrogen recycles sodium and thereby increases body weight by retaining water, whereas progesterone does the exact opposite, and both estrogen and progesterone thereby cause an electrolyte imbalance, measuring her first morning weight daily helped us identify her need for excess salt and water for her cycle prep, which in turn got rid of the hormonal migraines. She started with the LCHF diet and moved to ketogenic once feeling stable. During her marathon practices, and later during the actual races, the sugar gel packs were replaced with butter, cheese, and salt packs, and with the reduction of water from a cup at every stand to saltwater sips once in a while, she has been able to run marathons without ending with a migraine. She recently celebrated 1 year without a migraine.^1^

In another example, a teenager presented with her mom. He had cyclical vomiting and irritable bowel syndromes. Given his strong reaction to any form of carbohydrate, he started a specifically formulated high protein medium fat carnivore diet, which he has been able to maintain now for over 5 years. He and his parents celebrated his success of passing the Marine’s Crucible last year (see Footnote 1).

### 2.7 The low carbohydrate benefit

Why any of the low carbohydrate diets are beneficial is clear considering the carbohydrate sensitivity of the brain and the associated osmolality changes. As noted earlier, ketones are the preferred fuel for the brain that has glucose metabolism difficulties (192). Ketones in the brain are 3β-Hydroxybutyrate (3HB) and acetoacetate, which are fatty acids of medium chain triglycerides that can easily cross the blood brain barrier (193) and provide the fuel with great efficiency (194–196). Ketogenic diets have had great success with epileptic seizures (197), as well as Alzheimer’s disease, Multiple Sclerosis, and a host of other neurodegenerative diseases. The use of ketones by most brain functions defers the use of glucose to the glial cells (198). When we reduce glucose to the brain, we are also reducing glucose to the body. The ensuing fundamental changes lead to reversal of metabolic disease and re-establishment of insulin sensitivity in general, which improves the brain’s insulin sensitivity as well (181, 182, 199, 200).

The reversal and/or prevention of metabolic disease in the brain, especially in those populations whose brain cannot use glucose well as fuel, aids the healing processes of the brain. Further studies are needed in this area specifically with respect to migraine. These studies will help us to fully understand and underscore the numerous empirical success stories.

## 3 Discussion

Rather than medicating with strong brain modifying drugs in order to reduce the sensitivity of the brain of the migraineurs, why not modify the brain’s environment in such a way that the migraineur’s brain can retain plenty of sodium for the increased level of action potential it needs. Having enhanced sensory organs is not an absolute disadvantage. For example, we can frequently hear of stories where a migraineur smelled a gas leak, verified only by gas leak detection equipment, and saved a neighborhood. Medications blunt the hypersensory neurons of the migraine brain by blocking how the brain normally functions. While this may help reduce migraine symptoms, these medications degenerate the brain to work at a lower level of sensitivity. Instead of reducing the sensitivity, thereby dulling the senses of the migraineur, we could simply support the migraine brain with the right nutrients to reduce the chance for an electrolyte imbalance and the ensuing migraine.

The problem can be resolved by avoiding a high carbohydrate diet and by adding a sufficiently increased amount of salt to consumed water to increase blood volume, to provide enough sodium for the brain under any circumstance, so it can continuously support those important action potentials.

The ketogenic diet is specifically beneficial because it is a comfortable way of eating in a social setting, and it is also easy to remain on the ketogenic diet for a long time—perhaps for life. The production of ketones for the use of the brain has additional benefits, such as reversal of metabolic disease and the possible prevention/reversal of neurodegenerative diseases that often disproportionally afflict migraine sufferers (201, 202).

Finally, clinical trials are lacking in migraine research with nutrition. This is understandable, given how many medications migraine sufferers normally use, the subjectivity of evaluating if the diet reduced the number of migraines or migraine intensity, and the potential interactions between the many migraine medications taken and a given diet. To test the real benefit of a nutritional approach, the migraineurs would have to remain medication free during the trial and, of course, there is no placebo for food, so the control and trial groups would never be blinded, and thus inherently biased. In addition, since most migraines start in the morning hours—often the result of the dawn phenomenon blood glucose variations—the test subjects would have to be in a controlled environment for the entire length of a clinical trial. This is a hard task given that migraine prevalence is highest during childbearing and raring (203), making an in-house long-term clinical trial with meal and medication control very expensive and impractical.

## Data availability statement

The original contributions presented in the study are included in the article/supplementary material, further inquiries can be directed to the corresponding author.

## Author contributions

AS: Conceptualization, Visualization, Writing – original draft, Writing – review & editing.

## Footnotes

^1^https://stantonmigraineprotocol.com/testimonials/

## References

[1] Baigi K Stewart WF. Chapter 25 - headache and migraine: a leading cause of absenteeism In: Marcello L Margit LB, editors. Handbook of clinical neurology, vol. 131. Amsterdam Boston Heidelberg London New York Oxford Paris San Diego San Francisco Singapore Sydney Tokyo: Elsevier (2015). 447–63. 10.1016/B978-0-444-62627-1.00025-1 26563803

[2] Gawde P Shah H Patel H Bharathi KS Patel N Sethi Y. Revisiting Migraine: the evolving pathophysiology and the expanding management armamentarium. Cureus. (2023) 15:e34553. doi: 10.7759/cureus.34553, PMID: 36879707 PMC9985459

[3] Lampl C Versijpt J Amin FM Deligianni CI Gil-Gouveia R Jassal T. European headache federation (EHF) critical re-appraisal and meta-analysis of oral drugs in migraine prevention—part 1: amitriptyline. J Headache Pain. (2023) 24:39. doi: 10.1186/s10194-023-01573-6, PMID: 37038134 PMC10088191

[4] Bigal ME Krymchantowski AV. Emerging drugs for migraine prophylaxis and treatment. MedGenMed. (2006) 8:31. PMID: 16926770 PMC1785190

[5] Alpay K Ertaş M Orhan EK Üstay DK Lieners C Baykan B. Diet restriction in migraine, based on IgG against foods: a clinical double-blind, randomised, cross-over trial. Cephalalgia. (2010) 30:829–37. doi: 10.1177/0333102410361404, PMID: 20647174 PMC2899772

[6] Hoffmann J Schirra T Lo H Neeb L Reuter U Martus P. The influence of weather on migraine - are migraine attacks predictable? Ann Clin Transl Neurol. (2015) 2:22–8. doi: 10.1002/acn3.139, PMID: 25642431 PMC4301671

[7] Sacco S Ricci S Degan D Carolei A. Migraine in women: the role of hormones and their impact on vascular diseases. J Headache Pain. (2012) 13:177–89. doi: 10.1007/s10194-012-0424-y, PMID: 22367631 PMC3311830

[8] Warshaw LJ Lipton RB Silberstein SD. Migraine: a "woman's disease?". Women Health. (1998) 28:79–99. doi: 10.1300/J013v28n02_05, PMID: 10067807

[9] Lipton RB Bigal ME. Migraine: epidemiology, impact, and risk factors for progression. Headache. (2005) 45:S3–s13. doi: 10.1111/j.1526-4610.2005.4501001.x 15833088

[10] Tabrizi M Badeli H Hassanzadeh Rad A Aminzadeh V Shokuhifard A. Is infantile colic an early life expression of childhood Migraine? Iran J Child Neurol. (2017) 11:37–41. PMID: 28883875 PMC5582358

[11] Antonaci F Nappi G Galli F Manzoni GC Calabresi P Costa A. Migraine and psychiatric comorbidity: a review of clinical findings. J Headache Pain. (2011) 12:115–25. doi: 10.1007/s10194-010-0282-4 21210177 PMC3072482

[12] Bigal ME Kurth T Hu H Santanello N Lipton RB. Migraine and cardiovascular disease: possible mechanisms of interaction. Neurology. (2009) 72:1864–71. doi: 10.1212/WNL.0b013e3181a71220, PMID: 19470970 PMC2690985

[13] Avci AY Lakadamyali H Arikan S Benli US Kilinc M. High sensitivity C-reactive protein and cerebral white matter hyperintensities on magnetic resonance imaging in migraine patients. J Headache Pain. (2015) 16:9. doi: 10.1186/1129-2377-16-9, PMID: 25595197 PMC4417106

[14] Azimova JE Sergeev AV Korobeynikova LA Kondratieva NS Kokaeva ZG Shaikhaev GO. Effects of MTHFR gene polymorphism on the clinical and electrophysiological characteristics of migraine. BMC Neurol. (2013) 13:103. doi: 10.1186/1471-2377-13-103, PMID: 23915182 PMC3750291

[15] Benson MD Rebar RW. Relationship of migraine headache and stroke to oral contraceptive use. J Reprod Med. (1986) 31:1082–8. PMID: 3540297

[16] Øie LR Kurth T Gulati S Dodick DW. Migraine and risk of stroke. J Neurol Neurosurg Psychiatry. (2020) 91:593–604. doi: 10.1136/jnnp-2018-318254, PMID: 32217787 PMC7279194

[17] Bernecker C Pailer S Kieslinger P Horejsi R Möller R Lechner A. Increased matrix metalloproteinase activity is associated with migraine and migraine-related metabolic dysfunctions. Eur J Neurol. (2011) 18:571–6. doi: 10.1111/j.1468-1331.2010.03205.x, PMID: 20825467

[18] Bernecker C Ragginer C Fauler G Horejsi R Möller R Zelzer S. Oxidative stress is associated with migraine and migraine-related metabolic risk in females. Eur J Neurol. (2011) 18:1233–9. doi: 10.1111/j.1468-1331.2011.03414.x, PMID: 21518147

[19] Borkum JM. Migraine triggers and oxidative stress: a narrative review and synthesis. Headache: the journal of head and face. Pain. (2016) 56:12–35. doi: 10.1111/head.12725 26639834

[20] Biondi DM. Is migraine a neuropathic pain syndrome? Curr Pain Headache Rep. (2006) 10:167–78. doi: 10.1007/s11916-006-0042-y 18778570

[21] Blau JN Pyke DA. Effect of diabetes on migraine. Lancet. (1970) 2:740–2. doi: 10.1016/S0140-6736(70)92588-2, PMID: 4193691

[22] Borsook D Maleki N Becerra L McEwen B. Understanding Migraine through the Lens of maladaptive stress responses: a model disease of allostatic load. Neuron. (2012) 73:219–34. doi: 10.1016/j.neuron.2012.01.001 22284178

[23] Casucci G Villani V Cologno D D’Onofrio F. Migraine and metabolism. Neurol Sci. (2012) 33:81–5. doi: 10.1007/s10072-012-1047-4 22644177

[24] Cavestro C Rosatello A Micca G Ravotto M Marino MP Asteggiano G. Insulin metabolism is altered in migraineurs: a new pathogenic mechanism for migraine? Headache. (2007) 47:1436–42. doi: 10.1111/j.1526-4610.2007.00719.x, PMID: 18052953

[25] Amin FM Asghar MS Hougaard A Hansen AE Larsen VA de Koning PJH. Magnetic resonance angiography of intracranial and extracranial arteries in patients with spontaneous migraine without aura: a cross-sectional study. Lancet Neurol. (2013) 12:454–61. doi: 10.1016/S1474-4422(13)70067-X, PMID: 23578775

[26] Ambrosini A De Noordhout A Sándor PS Schoenen J. Electrophysiological studies in migraine: a comprehensive review of their interest and limitations. Cephalalgia. (2003) 23:13–31. doi: 10.1046/j.1468-2982.2003.00571.x 12699456

[27] Aurora S Cao Y Bowyer S Welch KMA. The occipital cortex is Hyperexcitable in Migraine: experimental evidence. Headache: the journal of head and face. Pain. (1999) 39:469–76. doi: 10.1046/j.1526-4610.1999.3907469.x 11279929

[28] Aurora SK Wilkinson F. The brain is hyperexcitable in migraine. Cephalalgia. (2007) 27:1442–53. doi: 10.1111/j.1468-2982.2007.01502.x 18034688

[29] Bashir A Lipton RB Ashina S Ashina M. Migraine and structural changes in the brain: a systematic review and meta-analysis. Neurology. (2013) 81:1260–8. doi: 10.1212/WNL.0b013e3182a6cb32, PMID: 23986301 PMC3795609

[30] Aurora SK Barrodale PM Tipton RL Khodavirdi A. Brainstem dysfunction in chronic Migraine as evidenced by neurophysiological and positron emission tomography studies*. Headache: the journal of head and face. Pain. (2007) 47:996–1003. doi: 10.1111/j.1526-4610.2007.00853.x 17635590

[31] Benemei S De Cesaris F Fusi C Rossi E Lupi C Geppetti P. TRPA1 and other TRP channels in migraine. J Headache Pain. (2013) 14:71. doi: 10.1186/1129-2377-14-71, PMID: 23941062 PMC3844362

[32] Burstein R. Deconstructing migraine headache into peripheral and central sensitization. Pain. (2001) 89:107–10. doi: 10.1016/S0304-3959(00)00478-4, PMID: 11166465

[33] Burstein R Noseda R Borsook D. Migraine: multiple processes, complex pathophysiology. J Neurosci. (2015) 35:6619–29. doi: 10.1523/JNEUROSCI.0373-15.2015, PMID: 25926442 PMC4412887

[34] Wolthausen J Sternberg S Gerloff C May A. Are cortical spreading depression and headache in migraine causally linked? Cephalalgia. (2009) 29:244–9. doi: 10.1111/j.1468-2982.2008.01713.x, PMID: 19025548

[35] D'Andrea G Gucciardi A Leon A. Elusive amines: migraine depends on biochemical abnormalities. Neurol Sci. (2022) 43:6299–304. doi: 10.1007/s10072-022-06241-2, PMID: 35840874

[36] Bowyer SM Aurora KS Moran JE Tepley N Welch KM. Magnetoencephalographic fields from patients with spontaneous and induced migraine aura. Ann Neurol. (2001) 50:582–7. doi: 10.1002/ana.1293, PMID: 11706963

[37] Charles AC Baca SM. Cortical spreading depression and migraine. Nat Rev Neurol. (2013) 9:637–44. doi: 10.1038/nrneurol.2013.192 24042483

[38] Tian D Izumi SI. Transcranial magnetic stimulation and neocortical neurons: the Micro-macro connection. Front Neurosci. (2022) 16:866245. doi: 10.3389/fnins.2022.866245, PMID: 35495053 PMC9039343

[39] Alaydin HC Vuralli D Keceli Y Can E Cengiz B Bolay H. Reduced short-latency afferent inhibition indicates impaired sensorimotor integrity during Migraine attacks. Headache. (2019) 59:906–14. doi: 10.1111/head.13554 31106418

[40] Dumkrieger G Chong CD Ross K Berisha V Schwedt TJ. Static and dynamic functional connectivity differences between migraine and persistent post-traumatic headache: a resting-state magnetic resonance imaging study. Cephalalgia. (2019) 39:1366–81. doi: 10.1177/0333102419847728, PMID: 31042064

[41] Chou BC Lerner A Barisano G Phung D Xu W Pinto SN. Functional MRI and diffusion tensor imaging in Migraine: a review of Migraine functional and White matter microstructural changes. J Cent Nerv Syst Dis. (2023) 15:11795735231205413. doi: 10.1177/11795735231205413, PMID: 37900908 PMC10612465

[42] Yang Y Peng X Chen Y. A case of migraine misdiagnosed as epilepsy. Acta Epileptol. (2023) 5:3. doi: 10.1186/s42494-022-00112-1 PMC11960377 40217377

[43] Mantegazza M Cestèle S. Pathophysiological mechanisms of migraine and epilepsy: similarities and differences. Neurosci Lett. (2018) 667:92–102. doi: 10.1016/j.neulet.2017.11.025, PMID: 29129678

[44] Lauritzen DJP Fabricius M Hartings JA Graf R Strong AJ. Clinical relevance of cortical spreading depression in neurological disorders: migraine, malignant stroke, subarachnoid and intracranial hemorrhage, and traumatic brain injury. J Cereb Blood Flow Metab. (2011) 31:17–35. doi: 10.1038/jcbfm.2010.191, PMID: 21045864 PMC3049472

[45] Blau JN. Migraine: theories of pathogenesis. Lancet. 339:1202–7. 10.1016/0140-6736(92)91140-4 1349944

[46] Chua AL Del Rio MS Silberstein S. Reference module in neuroscience and biobehavioral psychology Elsevier (2017).

[47] Cao Z Lai K-L Lin C-T Chuang C-H Chou C-C Wang S-J. Exploring resting-state EEG complexity before migraine attacks. Cephalalgia. 38:1296–306. doi: 10.1177/0333102417733953 28958151

[48] Society; TIH. The international classification of headache disorders 2013. (2013). [cited 2023 12/18/2023]. Available at: https://ichd-3.org/1-migraine/.

[49] Costandi M. Is migraine really gender specific? [internet]. Online: Medscape; (2023). (updated 12/12/2023; cited 2023 12/18/2023). Available at: https://www.medscape.com/viewarticle/migraine-really-female-disorder-2023a1000v35.

[50] Silberstein SD. Migraine [educational webpage]. Internet: Merck Manual; (2023). (cited 12/18/2023). Available at: https://www.merckmanuals.com/professional/neurologic-disorders/headache/migraine.

[51] Green M. 14 - headache In: Marshall RS Mayer SA, editors. On call neurology. 3. Amsterdam Boston Heidelberg London New York Oxford Paris San Diego San Francisco Singapore Sydney Tokyo: W.B. Saunders (2007). 175–92.

[52] Chen MH Sung YF Chien WC Chung CH Chen JW. Risk of Migraine after traumatic brain injury and effects of injury management levels and treatment modalities: a Nationwide population-based cohort study in Taiwan. J Clin Med. (2023) 12:1530. doi: 10.3390/jcm12041530 36836064 PMC9959615

[53] Youngson NA Morris MJ Ballard JWO. The mechanisms mediating the antiepileptic effects of the ketogenic diet, and potential opportunities for improvement with metabolism-altering drugs. Seizure. (2017) 52:15–9. doi: 10.1016/j.seizure.2017.09.005, PMID: 28941398

[54] Roehl K Falco-Walter J Ouyang B Balabanov A. Modified ketogenic diets in adults with refractory epilepsy: efficacious improvements in seizure frequency, seizure severity, and quality of life. Epilepsy Behav. (2019) 93:113–8. doi: 10.1016/j.yebeh.2018.12.010, PMID: 30867113

[55] Di Lorenzo C Ballerini G Barbanti P Bernardini A D’Arrigo G Egeo G. Applications of ketogenic diets in patients with headache: clinical recommendations. Nutri. (2021) 13:6. 10.3390/nu13072307 PMC8308539 34371817

[56] Martin-McGill KJ Jackson CF Bresnahan R Levy RG Cooper PN. Ketogenic diets for drug-resistant epilepsy. Cochrane Database Syst Rev. (2018) 11:Cd001903. doi: 10.1002/14651858.CD001903.pub4 30403286 PMC6517043

[57] Lester L McLaughlin S. SALT: a case for the Sodium Channel blockade Toxidrome and the mnemonic SALT. Ann Emerg Med. (2008) 51:214. doi: 10.1016/j.annemergmed.2007.09.028, PMID: 18206559

[58] Liamis G Liberopoulos E Barkas F Elisaf M. Diabetes mellitus and electrolyte disorders. World J Clin Cases. (2014) 2:488–96. doi: 10.12998/wjcc.v2.i10.488 25325058 PMC4198400

[59] Gankam Kengne F Decaux G. Hyponatremia and the brain. Kidney Int Rep. (2018) 3:24–35. doi: 10.1016/j.ekir.2017.08.015, PMID: 29340311 PMC5762960

[60] Arieff AI Guisado R. Effects on the central nervous system of hypernatremic and hyponatremic states. Kidney Int. (1976) 10:104–16. doi: 10.1038/ki.1976.82, PMID: 7702

[61] Holliday MA Kalayci MN Harrah J. Factors that limit brain volume changes in response to acute and sustained hyper- and hyponatremia. J Clin Invest. (1968) 47:1916–28. doi: 10.1172/JCI105882, PMID: 5666118 PMC297352

[62] Melton JE Patlak CS Pettigrew KD Cserr HF. Volume regulatory loss of Na, cl, and K from rat brain during acute hyponatremia. Am J Phys. (1987) 252:F661–9. doi: 10.1152/ajprenal.1987.252.4.F661 3565577

[63] Campbell DA Tonks EM Hay KM. An investigation of the salt and water balance in Migraine. Br Med J. (1951) 2:1424–9. doi: 10.1136/bmj.2.4745.1424 14879122 PMC2070698

[64] Arieff AI. Central nervous system manifestations of disordered sodium metabolism. Clin Endocrinol Metab. (1984) 13:269–94. doi: 10.1016/S0300-595X(84)80022-5, PMID: 6488574

[65] Hillier TA Abbott RD Barrett EJ. Hyponatremia: evaluating the correction factor for hyperglycemia. Am J Med. (1999) 106:399–403. doi: 10.1016/S0002-9343(99)00055-8, PMID: 10225241

[66] Ng PY Cheung RYT Ip A Chan WM Sin WC Yap DY. A retrospective cohort study on the clinical outcomes of patients admitted to intensive care units with dysnatremia. Sci Rep. (2023) 13:21236. doi: 10.1038/s41598-023-48399-5, PMID: 38040748 PMC10692105

[67] Holland-Bill L Christiansen CF Heide-Jørgensen U Ulrichsen SP Ring T Jørgensen JO. Hyponatremia and mortality risk: a Danish cohort study of 279508 acutely hospitalized patients. Eur J Endocrinol. (2015) 173:71–81. doi: 10.1530/EJE-15-0111, PMID: 26036812

[68] Yun G Baek SH Kim S. Evaluation and management of hypernatremia in adults: clinical perspectives. Korean J Intern Med. (2023) 38:290–302. doi: 10.3904/kjim.2022.346, PMID: 36578134 PMC10175862

[69] Adrogué HJ Madias NE. Hypernatremia. N Engl J Med. (2000) 342:1493–9. doi: 10.1056/NEJM200005183422006 10816188

[70] Kim SW. Hypernatemia: successful treatment. Electrolyte Blood Press. (2006) 4:66–71. doi: 10.5049/EBP.2006.4.2.66, PMID: 24459489 PMC3894528

[71] Strange K. Regulation of solute and water balance and cell volume in the central nervous system. J Am Soc Nephrol. (1992) 3:12–27. doi: 10.1681/ASN.V3112 1391705

[72] Sterns RH. Disorders of plasma sodium — causes, consequences, and correction. N Engl J Med. (2015) 372:55–65. doi: 10.1056/NEJMra1404489, PMID: 25551526

[73] Tso AR Trujillo A Guo CC Goadsby PJ Seeley WW. The anterior insula shows heightened interictal intrinsic connectivity in migraine without aura. Neurology. (2015) 84:1043–50. doi: 10.1212/WNL.0000000000001330 25663219 PMC4352101

[74] Hodkinson DJ Veggeberg R Kucyi A van Dijk KRA Wilcox SL Scrivani SJ. Cortico–cortical connections of primary sensory areas and associated symptoms in Migraine. eNeuro. (2016) 3:ENEURO.0163–16.2016. doi: 10.1523/ENEURO.0163-16.2016, PMID: 28101529 PMC5239993

[75] Liu H Ge H Xiang J Miao A Tang L Wu T. Resting state brain activity in patients with migraine: a magnetoencephalography study. J Headache Pain. (2015) 16:42. doi: 10.1186/s10194-015-0525-5, PMID: 25968099 PMC4429423

[76] Xue T Yuan K Zhao L Yu D Zhao L Dong T. Intrinsic brain network abnormalities in migraines without Aura revealed in resting-state fMRI. PLoS One. (2012) 7:e52927. doi: 10.1371/journal.pone.0052927, PMID: 23285228 PMC3532057

[77] Yu D Yuan K Luo L Zhai J Bi Y Xue T. Abnormal functional integration across core brain networks in migraine without aura. Mol Pain. (2017) 13:174480691773746. doi: 10.1177/1744806917737461 PMC5644367 28969471

[78] Schwedt TJ. Multisensory integration in Migraine. Curr Opin Neurol. (2013) 26:248–53. doi: 10.1097/WCO.0b013e328360edb1 23591684 PMC4038337

[79] Kaas JH. The evolution of complex sensory systems in mammals. J Exp Biol. (1989) 146:165–76. doi: 10.1242/jeb.146.1.165 2689560

[80] Sharma A Kumar R Aier I Semwal R Tyagi P Varadwaj P. Sense of smell: structural, functional, mechanistic advancements and challenges in human olfactory research. Curr Neuropharmacol. (2019) 17:891–911. doi: 10.2174/1570159X17666181206095626, PMID: 30520376 PMC7052838

[81] Antonaci F Voiticovschi-Iosob C Di Stefano AL Galli F Ozge A Balottin U. The evolution of headache from childhood to adulthood: a review of the literature. J Headache Pain. (2014) 15:15. doi: 10.1186/1129-2377-15-15, PMID: 24641507 PMC3995299

[82] Eren-Koçak E Dalkara T. Ion Channel dysfunction and Neuroinflammation in Migraine and depression. Front Pharmacol. (2021) 12:777607. doi: 10.3389/fphar.2021.777607, PMID: 34858192 PMC8631474

[83] Asbury C Rieke F Hille B Bothwell M Tuthill J. Physiology. Washington: University of Washington (2023).

[84] Coppola G Pierelli F Schoenen J. Is the cerebral cortex Hyperexcitable or Hyperresponsive in Migraine? Cephalalgia. (2007) 27:1427–39. doi: 10.1111/j.1468-2982.2007.01500.x 18034686

[85] Sihn D Kim S-P. A spike train distance robust to firing rate changes based on the earth Mover’s distance. Front Comput Neurosci. (2019) 13. doi: 10.3389/fncom.2019.00082, PMID: 31920607 PMC6914768

[86] Waxman SG Melker RJ. Closely spaced nodes of Ranvier in the mammalian brain. Brain Res. (1971) 32:445–8. doi: 10.1016/0006-8993(71)90337-4, PMID: 5134587

[87] Ritchie JM Rogart RB. Density of sodium channels in mammalian myelinated nerve fibers and nature of the axonal membrane under the myelin sheath. Proc Natl Acad Sci USA. (1977) 74:211–5. doi: 10.1073/pnas.74.1.211, PMID: 299947 PMC393228

[88] Cohen CCH Popovic MA Klooster J Weil MT Möbius W Nave KA. Saltatory conduction along myelinated axons involves a Periaxonal Nanocircuit. Cell. (2020) 180:311–22.e15. doi: 10.1016/j.cell.2019.11.039, PMID: 31883793 PMC6978798

[89] Baccus SA Burrell BD Sahley CL Muller KJ. Action potential reflection and failure at axon branch points cause stepwise changes in EPSPs in a neuron essential for learning. J Neurophysiol. (2000) 83:1693–700. doi: 10.1152/jn.2000.83.3.1693, PMID: 10712489

[90] Gemes G Koopmeiners A Rigaud M Lirk P Sapunar D Bangaru ML. Failure of action potential propagation in sensory neurons: mechanisms and loss of afferent filtering in C-type units after painful nerve injury. J Physiol. (2013) 591:1111–31. doi: 10.1113/jphysiol.2012.242750, PMID: 23148321 PMC3591718

[91] Meylakh N Henderson LA. Exploring alterations in sensory pathways in migraine. J Headache Pain. (2022) 23:5. doi: 10.1186/s10194-021-01371-y, PMID: 35021998 PMC8903612

[92] Goadsby PJ Holland PR Martins-Oliveira M Hoffmann J Schankin C Akerman S. Pathophysiology of Migraine: a disorder of sensory processing. Physiol Rev. (2017) 97:553–622. doi: 10.1152/physrev.00034.2015, PMID: 28179394 PMC5539409

[93] Rogawski MA. Common pathophysiologic mechanisms in Migraine and epilepsy. Arch Neurol. (2008) 65:709–14. doi: 10.1001/archneur.65.6.709, PMID: 18541791

[94] Pusic AD Mitchell HM Kunkler PE Klauer N Kraig RP. Spreading depression transiently disrupts myelin via interferon-gamma signaling. Exp Neurol. (2015) 264:43–54. doi: 10.1016/j.expneurol.2014.12.001, PMID: 25500111 PMC4324018

[95] Alizadeh A Dyck SM Karimi-Abdolrezaee S. Myelin damage and repair in pathologic CNS: challenges and prospects. Front Mol Neurosci. (2015) 8:35. doi: 10.3389/fnmol.2015.00035 26283909 PMC4515562

[96] Pietrobon D. Calcium channels and migraine. Biochimica et Biophysica Acta (BBA). Biomembranes. (2013) 1828:1655–65. doi: 10.1016/j.bbamem.2012.11.012 23165010

[97] Lee J-Y Kim M. Current issues in Migraine genetics. J Clin Neurol. (2005) 1:8–13. doi: 10.3988/jcn.2005.1.1.8, PMID: 20396468 PMC2854934

[98] Surtees R. Inherited ion channel disorders. Eur J Pediatr. (2000) 159:S199–203. doi: 10.1007/PL00014403 11216900

[99] Catterall WA Dib-Hajj S Meisler MH Pietrobon D. Inherited neuronal ion Channelopathies: new windows on complex neurological diseases. J Neurosci. (2008) 28:11768–77. doi: 10.1523/JNEUROSCI.3901-08.2008, PMID: 19005038 PMC3177942

[100] Kim J-B. Channelopathies. Korean J Pediatr. (2014) 57:1–18. doi: 10.3345/kjp.2014.57.1.1, PMID: 24578711 PMC3935107

[101] Maggioni F Mainardi F Dainese F Lisotto C Zanchin G. Migraine secondary to superior oblique Myokymia. Cephalalgia. (2007) 27:1283–5. doi: 10.1111/j.1468-2982.2007.01422.x, PMID: 17692104

[102] Levin M Ward TN. Ophthalmoplegic migraine. Curr Pain Headache Rep. (2004) 8:306–9. doi: 10.1007/s11916-004-0013-0 15228891

[103] Rose MR. Neurological channelopathies. BMJ. (1998) 316:1104–5. doi: 10.1136/bmj.316.7138.1104, PMID: 9552942 PMC1112934

[104] Staehr C Aalkjaer C Matchkov VV. The vascular Na,K-ATPase: clinical implications in stroke, migraine, and hypertension. Clin Sci. (2023) 137:1595–618. doi: 10.1042/CS20220796, PMID: 37877226 PMC10600256

[105] Science WIo. The human gene database internet: Weizmann Institute of Science; 2017 [cited 2017 1/28/2017]. The human genome database]. Available at: http://www.genecards.org.

[106] Zukin RS Jover T Yokota H Calderone A Simionescu M Lau CG. Chapter 42 - molecular and cellular mechanisms of ischemia-induced neuronal death In: Mohr JP Choi DW Grotta JC Weir B Wolf PA, editors. Stroke. Fourth ed. Philadelphia: Churchill Livingstone (2004). 829–54.

[107] Ayata C Lauritzen M. Spreading depression, spreading depolarizations, and the cerebral vasculature. Physiol Rev. (2015) 95:953–93. doi: 10.1152/physrev.00027.2014, PMID: 26133935 PMC4491545

[108] Enger R Tang W Vindedal GF Jensen V Johannes Helm P Sprengel R. Dynamics of ionic shifts in cortical spreading depression. Cereb Cortex. (2015) 25:4469–76. doi: 10.1093/cercor/bhv054, PMID: 25840424 PMC4816793

[109] Mantantzis K Schlaghecken F Sünram-Lea SI Maylor EA. Sugar rush or sugar crash? A meta-analysis of carbohydrate effects on mood. Neurosci Biobehav Rev. (2019) 101:45–67. doi: 10.1016/j.neubiorev.2019.03.016 30951762

[110] Longo DL Fauci AS Kasper DL Hauser SL Jameson JL Loscalzo J. Harrison's manual of medicine. 18th ed. New York: McGraw Hill Medical (2013).

[111] Haigh S Karanovic O Wilkinson F Wilkins A. Cortical hyperexcitability in migraine and aversion to patterns. Cephalalgia. (2012) 32:236–40. doi: 10.1177/0333102411433301, PMID: 22234882 PMC4011802

[112] Antal A Arlt S Nitsche M Chadaide Z Paulus W. Higher variability of Phosphene thresholds in Migraineurs than in controls: a consecutive transcranial magnetic stimulation study. Cephalalgia. (2006) 26:865–70. doi: 10.1111/j.1468-2982.2006.01132.x, PMID: 16776703

[113] Nardone R Brigo F Trinka E. Acute symptomatic seizures caused by electrolyte disturbances. J Clin Neurol (Seoul, Korea). (2016) 12:21–33. doi: 10.3988/jcn.2016.12.1.21, PMID: 26754778 PMC4712283

[114] Schwartzkroin PA Baraban SC Hochman DW. Osmolarity, ionic flux, and changes in brain excitability. Epilepsy Res. (1998) 32:275–85. doi: 10.1016/S0920-1211(98)00058-8, PMID: 9761327

[115] Hunter RW Bailey MA. Hyperkalemia: pathophysiology, risk factors and consequences. Nephrol Dial Transplant. (2019) 34:iii2–iii11. doi: 10.1093/ndt/gfz206 31800080 PMC6892421

[116] Alt KW Al-Ahmad A Woelber JP. Nutrition and health in human evolution-past to present. Nutrients. (2022) 14. doi: 10.3390/nu14173594, PMID: 36079850 PMC9460423

[117] Hertz L Rothman DL. Glucose, lactate, β-Hydroxybutyrate, acetate, GABA, and succinate as substrates for synthesis of glutamate and GABA in the glutamine–glutamate/GABA cycle. In: Schousboe A Sonnewald U, editors. The glutamate/GABA-glutamine cycle: Amino acid neurotransmitter homeostasis. Cham: Springer International Publishing; (2016). p. 9–42. PMID: 10.1007/978-3-319-45096-4_2 27885625

[118] Malkov A Ivanov AI Popova I Mukhtarov M Gubkina O Waseem T. Reactive oxygen species initiate a metabolic collapse in hippocampal slices: potential trigger of cortical spreading depression. J Cereb Blood Flow Metab. (2014) 34:1540–9. doi: 10.1038/jcbfm.2014.121, PMID: 25027308 PMC4158675

[119] Pietrobon D Moskowitz MA. Chaos and commotion in the wake of cortical spreading depression and spreading depolarizations. Nat Rev Neurosci. (2014) 15:379–93. doi: 10.1038/nrn3770, PMID: 24857965

[120] Smith JM Bradley DP James MF Huang CL. Physiological studies of cortical spreading depression. Biol Rev Camb Philos Soc. (2006) 81:457. doi: 10.1017/S1464793106007081 16848916

[121] Somjen GG. Mechanisms of spreading depression and hypoxic spreading depression-like depolarization. Physiol Rev. (2001) 81:1065–96. doi: 10.1152/physrev.2001.81.3.1065, PMID: 11427692

[122] Ayata C. Pearls and pitfalls in experimental models of spreading depression. Cephalalgia. (2013) 33:604–13. doi: 10.1177/0333102412470216, PMID: 23671256

[123] Yen S Wu HY Wang Y Huang CM Wu CW Chen JH. Revisiting the effects of exercise on cerebral neurovascular functions in rats using multimodal assessment techniques. iScience. (2023) 26:106354. doi: 10.1016/j.isci.2023.106354, PMID: 37035001 PMC10074158

[124] Poff AM Moss S Soliven M D'Agostino DP. Ketone supplementation: meeting the needs of the brain in an energy crisis. Front Nutr. (2021) 8:8. doi: 10.3389/fnut.2021.783659 PMC8734638 35004814

[125] Nakazawa M Kodama S Matsuo T. Effects of ketogenic diet on electroconvulsive threshold and brain contents of adenosine nucleotides. Brain and Development. (1983) 5:375–80. doi: 10.1016/S0387-7604(83)80042-4, PMID: 6638394

[126] Herrera E Amusquivar E. Lipid metabolism in the fetus and the newborn. Diabetes Metab Res Rev. (2000) 16:202–10. doi: 10.1002/1520-7560(200005/06)16:3<202::AID-DMRR116>3.0.CO;2-# 10867720

[127] Herrera E. Lipid metabolism in pregnancy and its consequences in the fetus and newborn. Endocrine. (2002) 19:43–56. doi: 10.1385/ENDO:19:1:43 12583601

[128] Storlien L Oakes ND Kelley DE. Metabolic flexibility. Proc Nutr Soc. (2007) 63:363–8. doi: 10.1079/PNS2004349 15294056

[129] Pogoda JM Gross NB Arakaki X Fonteh AN Cowan RP Harrington MG. Severe headache or Migraine history is inversely correlated with dietary sodium intake: NHANES 1999–2004. Headache. (2016) 56:688–98. doi: 10.1111/head.12792 27016121 PMC4836999

[130] Blitshteyn S. Dietary sodium intake and Migraine: is salt the answer? Headache: the journal of head and face. Pain. (2016) 56:1210–1. doi: 10.1111/head.12869 27432625

[131] Haghdoost F. Is there an inverse relationship between Migraine and dietary sodium intake? Headache: the journal of head and face. Pain. (2016) 56:1212–3. doi: 10.1111/head.12848 27432626

[132] Kodde IF van der Stok SRT de Jong JW. Metabolic and genetic regulation of cardiac energy substrate preference. Comp Biochem Physiol -Part A Mol Integr Physiol. (2007) 146:26–39. doi: 10.1016/j.cbpa.2006.09.014 17081788

[133] Jensen MD. Fate of fatty acids at rest and during exercise: regulatory mechanisms. Acta Physiol Scand. (2003) 178:385–90. doi: 10.1046/j.1365-201X.2003.01167.x, PMID: 12864743

[134] Smith T Gerich JE. Glucagon secretion, regulation of In: Henry HL Norman AW, editors. Encyclopedia of hormones. New York: Academic Press (2003). 74–82.

[135] Lindsay DB. Fatty acids as energy sources. Proc Nutr Soc. (1975) 34:241–8. doi: 10.1079/PNS19750045 1108029

[136] Haslam RL Bezzina A Herbert J Spratt N Rollo ME Collins CE. Can ketogenic diet therapy improve Migraine frequency, severity and duration? Healthcare (Basel). (2021) 9. doi: 10.3390/healthcare9091105 PMC8471252 34574879

[137] Tereshko Y Dal Bello S Di Lorenzo C Pez S Pittino A Sartor R. 2:1 ketogenic diet and low-glycemic-index diet for the treatment of chronic and episodic migraine: a single-center real-life retrospective study. J Headache Pain. (2023) 24:95. doi: 10.1186/s10194-023-01635-9, PMID: 37501109 PMC10375678

[138] Pogoda JM Gross NB Arakaki X Fonteh AN Cowan RP Harrington MG. Severe headache or Migraine history is inversely correlated with dietary sodium intake: NHANES 1999-2004: a response. Headache. (2016) 56:1216–8. doi: 10.1111/head.12868 27432628

[139] Bogie JFJ Haidar M Kooij G Hendriks JJA. Fatty acid metabolism in the progression and resolution of CNS disorders. Adv Drug Deliv Rev. (2020) 159:198–213. doi: 10.1016/j.addr.2020.01.004, PMID: 31987838

[140] Altayyar M Nasser JA Thomopoulos D Bruneau M Jr. The implication of physiological ketosis on the cognitive brain: a narrative review. Nutrients. (2022) 14. doi: 10.3390/nu14030513, PMID: 35276871 PMC8840718

[141] Barañano KW Hartman AL. The ketogenic diet: uses in epilepsy and other neurologic illnesses. Curr Treat Options Neurol. (2008) 10:410–9. doi: 10.1007/s11940-008-0043-8, PMID: 18990309 PMC2898565

[142] Stafstrom CE Rho JM. The ketogenic diet as a treatment paradigm for diverse neurological disorders. Front Pharmacol. (2012) 3:59. 22509165 10.3389/fphar.2012.00059 PMC3321471

[143] Edmond J Robbins RA Bergstrom JD Cole RA de Vellis J. Capacity for substrate utilization in oxidative metabolism by neurons, astrocytes, and oligodendrocytes from developing brain in primary culture. J Neurosci Res. (1987) 18:551–61. doi: 10.1002/jnr.490180407, PMID: 3481403

[144] Ruan Y Chen L She D Chung Y Ge L Han L. Ketogenic diet for epilepsy: an overview of systematic review and meta-analysis. Eur J Clin Nutr. (2022) 76:1234–44. doi: 10.1038/s41430-021-01060-8, PMID: 35027683

[145] Neri LCL Ferraris C Catalano G Guglielmetti M Pasca L Pezzotti E. Ketosis and migraine: a systematic review of the literature and meta-analysis. Front Nutr. (2023) 10:10. doi: 10.3389/fnut.2023.1204700 PMC10292926 37377485

[146] Jensen NJ Wodschow HZ Nilsson M Rungby J. Effects of ketone bodies on brain metabolism and function in neurodegenerative diseases. Int J Mol Sci. (2020) 21. doi: 10.3390/ijms21228767, PMID: 33233502 PMC7699472

[147] Yeo WK Carey AL Burke L Spriet LL Hawley JA. Fat adaptation in well-trained athletes: effects on cell metabolism. Appl Physiol Nutr Metab. (2011) 36:12–22. doi: 10.1139/H10-089, PMID: 21326374

[148] Harvey CJC Schofield GM Zinn C Thornley S. Effects of differing levels of carbohydrate restriction on mood achievement of nutritional ketosis, and symptoms of carbohydrate withdrawal in healthy adults: a randomized clinical trial. Nutrition. (2019) 67-68:100005. doi: 10.1016/j.nutx.2019.100005, PMID: 34332710

[149] Bostock ECS Kirkby KC Taylor BV Hawrelak JA. Consumer reports of "keto flu" associated with the ketogenic diet. Front Nutr. (2020) 7:20. doi: 10.3389/fnut.2020.00020, PMID: 32232045 PMC7082414

[150] DeFronzo RA. The effect of insulin on renal sodium metabolism. A review with clinical implications. Diabetologia. (1981) 21:165–71. doi: 10.1007/BF00252649, PMID: 7028550

[151] Tan-Shalaby J. Ketogenic diets and Cancer: emerging evidence. Fed Pract. (2017) 34:37s–42s. doi: 10.12788/fp.0457 PMC6375425 30766299

[152] Kolanowski J. Influence of insulin and glucagon on sodium balance in obese subjects during fasting and refeeding. Int J Obes. (1981) 5:105–14. 6113218

[153] Barrea L Verde L Santangeli P Lucà S Docimo A Savastano S. Very low-calorie ketogenic diet (VLCKD): an antihypertensive nutritional approach. J Transl Med. (2023) 21:128. doi: 10.1186/s12967-023-03956-4, PMID: 36800966 PMC9936635

[154] Poplawski MM Mastaitis JW Isoda F Grosjean F Zheng F Mobbs CV. Reversal of diabetic nephropathy by a ketogenic diet. PLoS One. (2011) 6:e18604. doi: 10.1371/journal.pone.0018604, PMID: 21533091 PMC3080383

[155] Alarim RA Alasmre FA Alotaibi HA Alshehri MA Hussain SA. Effects of the ketogenic diet on glycemic control in diabetic patients: Meta-analysis of clinical trials. Cureus. (2020) 12:e10796. doi: 10.7759/cureus.10796, PMID: 33163300 PMC7641470

[156] Cox N Gibas S Salisbury M Gomer J Gibas K. Ketogenic diets potentially reverse type II diabetes and ameliorate clinical depression: a case study. Diabetes Metab Syndr. (2019) 13:1475–9. doi: 10.1016/j.dsx.2019.01.055, PMID: 31336509

[157] Hallberg SJ McKenzie AL Williams PT Bhanpuri NH Peters AL Campbell WW. Effectiveness and safety of a novel care model for the management of type 2 diabetes at 1 year: an open-label, non-randomized, controlled study. Diab Ther. (2018) 9:613–21. doi: 10.1007/s13300-018-0386-4 PMC6104276 29508274

[158] Millard-Stafford M Snow TK Jones ML Suh H. The beverage hydration index: influence of electrolytes, carbohydrate and protein. Nutrients. (2021) 13. doi: 10.3390/nu13092933, PMID: 34578811 PMC8465972

[159] Blaustein MP Leenen FHH Chen L Golovina VA Hamlyn JM Pallone TL. How NaCl raises blood pressure: a new paradigm for the pathogenesis of salt-dependent hypertension. Am J Physiol Heart Circ Physiol. (2012) 302:H1031–49. doi: 10.1152/ajpheart.00899.2011 22058154 PMC3311458

[160] Tietjen GE Herial NA Hardgrove J Utley C White L. Migraine comorbidity constellations. Headache. (2007) 47:857–65. doi: 10.1111/j.1526-4610.2007.00814.x, PMID: 17578536

[161] Kurth T. Associations between migraine and cardiovascular disease. Expert Rev Neurother. (2007) 7:1097–104. doi: 10.1586/14737175.7.9.1097 17868009

[162] Schürks M Rist PM Bigal ME Buring JE Lipton RB Kurth T. Migraine and cardiovascular disease: systematic review and meta-analysis. Brit Med J. (2009) 339:b3914. doi: 10.1136/bmj.b3914, PMID: 19861375 PMC2768778

[163] Stanton AA. Are we sure we know the risk factors for cardiovascular disease?∗. J Am Coll Cardiol. (2023) 81:2255–7. doi: 10.1016/j.jacc.2023.04.012, PMID: 37286255

[164] Trelle S Reichenbach S Wandel S Hildebrand P Tschannen B Villiger PM. Cardiovascular safety of non-steroidal anti-inflammatory drugs: network meta-analysis. BMJ. (2011) 342:c7086. doi: 10.1136/bmj.c7086, PMID: 21224324 PMC3019238

[165] Ji Y Chen S Wang Q Xiang B Xu Z Zhong L. Intolerable side effects during propranolol therapy for infantile hemangioma: frequency, risk factors and management. Sci Rep. (2018) 8:4264. doi: 10.1038/s41598-018-22787-8, PMID: 29523832 PMC5844887

[166] Hall GC Brown MM Mo J MacRae KD. Triptans in migraine: the risks of stroke, cardiovascular disease, and death in practice. Neurology. (2004) 62:563–8. doi: 10.1212/01.WNL.0000110312.36809.7F 14981171

[167] Yekehtaz H Farokhnia M Akhondzadeh S. Cardiovascular considerations in antidepressant therapy: an evidence-based review. J Tehran Heart Cent. (2013) 8:169–76. PMID: 26005484 PMC4434967

[168] Favoni V Giani L Al-Hassany L Asioli GM Butera C de Boer I. CGRP and migraine from a cardiovascular point of view: what do we expect from blocking CGRP? J Headache Pain. (2019) 20:27. doi: 10.1186/s10194-019-0979-y, PMID: 30866804 PMC6734543

[169] MaassenVanDenBrink A Meijer J Villalón CM Ferrari MD. Wiping out CGRP: potential cardiovascular risks. Trends Pharmacol Sci. (2016) 37:779–88. doi: 10.1016/j.tips.2016.06.002, PMID: 27338837

[170] Mohammadianinejad SE Abbasi V Sajedi SA Majdinasab N Abdollahi F Hajmanouchehri R. Zonisamide versus topiramate in migraine prophylaxis: a double-blind randomized clinical trial. Clin Neuropharmacol. (2011) 34:174–7. doi: 10.1097/WNF.0b013e318225140c, PMID: 21738025

[171] Mente A O'Donnell M Rangarajan S Dagenais G Lear S McQueen M. Associations of urinary sodium excretion with cardiovascular events in individuals with and without hypertension: a pooled analysis of data from four studies. Lancet. (2016) 388:465–75. doi: 10.1016/S0140-6736(16)30467-6, PMID: 27216139

[172] DiNicolantonio JJ O'Keefe JH. Sodium restriction and insulin resistance: a review of 23 clinical trials. J Metab Health. (2023) 6:a78. doi: 10.4102/jir.v6i1.78

[173] Garg R Williams GH Hurwitz S Brown NJ Hopkins PN Adler GK. Low-salt diet increases insulin resistance in healthy subjects. Metabolism. (2011) 60:965–8. doi: 10.1016/j.metabol.2010.09.005, PMID: 21036373 PMC3036792

[174] Garg R Sun B Williams J. Effect of low salt diet on insulin resistance in salt-sensitive versus salt-resistant hypertension. Hypertension. (2014) 64:1384–7. doi: 10.1161/HYPERTENSIONAHA.114.03880, PMID: 25185125 PMC4230999

[175] Seçil Y Ünde C Beckmann YY Bozkaya YT Özerkan F Başoğlu M. Blood pressure changes in Migraine patients before, during and after Migraine attacks. Pain Pract. (2010) 10:222–7. doi: 10.1111/j.1533-2500.2009.00349.x, PMID: 20158621

[176] Cianchetti C Hmaidan Y Finco G Ledda MG. Scalp periarterial saline efficacy in migraine and relation to exploding and imploding headache. J Neurol. (2009) 256:1109–13. doi: 10.1007/s00415-009-5077-7, PMID: 19252768

[177] Gupta S Oosthuizen R Pulfrey S. Treatment of acute migraine in the emergency department. Can Fam Physician. (2014) 60:47–9. PMID: 24452560 PMC3994811

[178] Ali AS Stillman M. What inpatient treatments do we have for acute intractable migraine? Cleve Clin J Med. (2018) 85:514–6. doi: 10.3949/ccjm.85a.17049, PMID: 30004374

[179] Imamura M Sasaki H Hayashi K Shibata S. Mid-point of the active phase is better to achieve the natriuretic effect of acute salt load in mice. Nutrients. (2023) 15. doi: 10.3390/nu15071679, PMID: 37049519 PMC10096866

[180] Negussie AB Dell AC Davis BA Geibel JP. Colonic fluid and electrolyte transport 2022: an update. Cells. (2022) 11. doi: 10.3390/cells11101712, PMID: 35626748 PMC9139964

[181] Ebbeling CB Feldman HA Klein GL Wong JMW Bielak L Steltz SK. Effects of a low carbohydrate diet on energy expenditure during weight loss maintenance: randomized trial. BMJ. (2018) 363:k4583. doi: 10.1136/bmj.k4583 30429127 PMC6233655

[182] Ludwig DS Ebbeling CB. The carbohydrate-insulin model of obesity: beyond "calories in, calories out". JAMA Intern Med. (2018) 178:1098–103. doi: 10.1001/jamainternmed.2018.2933, PMID: 29971406 PMC6082688

[183] Nordmann AJ Nordmann A Briel M Keller U Yancy WS Brehm BJ. Effects of low-carbohydrate vs low-fat diets on weight loss and cardiovascular risk factors: a meta-analysis of randomized controlled trials. Arch Intern Med. (2006) 166:285–93. doi: 10.1001/archinte.166.3.285, PMID: 16476868

[184] Paoli A Rubini A Volek JS Grimaldi KA. Beyond weight loss: a review of the therapeutic uses of very-low-carbohydrate (ketogenic) diets. Eur J Clin Nutr. (2013) 67:789–96. doi: 10.1038/ejcn.2013.116, PMID: 23801097 PMC3826507

[185] Noakes TD Windt J. Evidence that supports the prescription of low-carbohydrate high-fat diets: a narrative review. Br J Sports Med. (2017) 51:133–9. doi: 10.1136/bjsports-2016-096491, PMID: 28053201

[186] Feinman RD Pogozelski WK Astrup A Bernstein RK Fine EJ Westman EC. Dietary carbohydrate restriction as the first approach in diabetes management: critical review and evidence base. Nutrition. (2015) 31:1–13. doi: 10.1016/j.nut.2014.06.011, PMID: 25287761

[187] McKenzie A Hallberg S Creighton BC Volk BM Link T Abner M. A novel intervention including individualized nutritional recommendations reduces hemoglobin A1c level, medication use, and weight in type 2 diabetes. JMIR Diabetes. (2017) 2:2. doi: 10.2196/diabetes.6981 PMC6238887 30291062

[188] Valente M Garbo R Filippi F Antonutti A Ceccarini V Tereshko Y. Migraine prevention through ketogenic diet: more than body mass composition changes. J Clin Med. (2022) 11. doi: 10.3390/jcm11174946, PMID: 36078876 PMC9456603

[189] Razeghi Jahromi S Ghorbani Z Martelletti P Lampl C Togha M. Association of diet and headache. J Headache Pain. (2019) 20:106. doi: 10.1186/s10194-019-1057-1, PMID: 31726975 PMC6854770

[190] Fortini I Felsenfeld Junior BD. Headaches and obesity. Arq Neuropsiquiatr. (2022) 80:204–13. doi: 10.1590/0004-282x-anp-2022-s106, PMID: 35976296 PMC9491411

[191] Del Moro L Rota E Pirovano E Rainero I. Migraine, brain glucose metabolism and the "Neuroenergetic" hypothesis: a scoping review. J Pain. (2022) 23:1294–317. doi: 10.1016/j.jpain.2022.02.006, PMID: 35296423

[192] LaManna JC Salem N Puchowicz M Erokwu B Koppaka S Flask C. Ketones suppress brain glucose consumption. Adv Exp Med Biol. (2009) 645:301–6. doi: 10.1007/978-0-387-85998-9_45, PMID: 19227486 PMC2874681

[193] Pardridge WM. Blood-brain barrier transport of glucose, free fatty acids, and ketone bodies In: Vranic M Efendic S Hollenberg CH, editors. Fuel homeostasis and the nervous system. Boston, MA: Springer US (1991). 43–53. 10.1007/978-1-4684-5931-9_5 1927689

[194] Newman JC Verdin E. Ketone bodies as signaling metabolites. Trends Endocrinol Metab. (2014) 25:42–52. doi: 10.1016/j.tem.2013.09.002, PMID: 24140022 PMC4176946

[195] García-Rodríguez D Giménez-Cassina A. Ketone bodies in the brain beyond fuel metabolism: from excitability to gene expression and cell signaling. Front Mol Neurosci. (2021) 14:14. doi: 10.3389/fnmol.2021.732120 PMC8429829 34512261

[196] Sokoloff L. Metabolism of ketone bodies by the brain. Annu Rev Med. (1973) 24:271–80. doi: 10.1146/annurev.me.24.020173.001415 4575857

[197] Kim DY Simeone KA Simeone TA Pandya JD Wilke JC Ahn Y. Ketone bodies mediate anti-seizure effects through mitochondrial permeability transition. Ann Neurol. (2015) 78:77–87. doi: 10.1002/ana.24424, PMID: 25899847 PMC4480159

[198] Zhang Y Kuang Y Xu K Harris D Lee Z LaManna J. Ketosis proportionately spares glucose utilization in brain. J Cereb Blood Flow Metab. (2013) 33:1307–11. doi: 10.1038/jcbfm.2013.87, PMID: 23736643 PMC3734783

[199] Pogozelski W Arpaia N Priore S. The metabolic effects of low-carbohydrate diets and incorporation into a biochemistry course. Biochem Mol Biol Educ. (2005) 33:91–100. doi: 10.1002/bmb.2005.494033022445, PMID: 21638552

[200] O’Neill BJ. Effect of low-carbohydrate diets on cardiometabolic risk, insulin resistance, and metabolic syndrome. Curr Opin Endocrinol Diabetes Obes. (2020) 27:301–7. doi: 10.1097/MED.0000000000000569, PMID: 32773574

[201] Kim J Ha WS Park SH Han K Baek MS. Association between migraine and Alzheimer's disease: a nationwide cohort study. Front Aging Neurosci. (2023) 15:1196185. doi: 10.3389/fnagi.2023.1196185, PMID: 37304073 PMC10248237

[202] Morton RE St John PD Tyas SL. Migraine and the risk of all-cause dementia, Alzheimer's disease, and vascular dementia: a prospective cohort study in community-dwelling older adults. Int J Geriatr Psychiatry. (2019) 34:1667–76. doi: 10.1002/gps.5180, PMID: 31486140

[203] Victor T Hu X Campbell J Buse D Lipton R. Migraine prevalence by age and sex in the United States: a life-span study. Cephalalgia. (2010) 30:1065–72. doi: 10.1177/0333102409355601, PMID: 20713557

